# Association of polymorphisms in *MALAT1* with the risk of endometrial cancer in Southern Chinese women

**DOI:** 10.1002/jcla.23146

**Published:** 2019-12-27

**Authors:** Guange Chen, Mingyao Zhang, Zongwen Liang, Sailing Chen, Feng Chen, Jiawei Zhu, Manman Zhao, Jing He, Wenfeng Hua, Ping Duan

**Affiliations:** ^1^ Department of Obstetrics and Gynecology The Second Affiliated Hospital and Yuying Children's Hospital of Wenzhou Medical University Wenzhou China; ^2^ Department of Pediatric Surgery Guangzhou Women and Children's Medical Center Guangzhou Institute of Pediatrics Guangzhou Medical University Guangzhou China; ^3^ Department of Laboratory Medicine and Central Laboratories Guangdong Second Provincial General Hospital Guangzhou China

**Keywords:** endometrial cancer, *MALAT1*, risk, single nucleotide polymorphisms

## Abstract

**Background:**

Endometrial cancer is the most common gynecologic malignancy worldwide. Polymorphisms in *MALAT1* have been demonstrated to play critical roles in cancer. However, the roles of *MALAT1* polymorphisms in the etiology of endometrial cancer have not been well documented.

**Methods:**

We genotyped three *MALAT1* polymorphisms in 249 endometrial cancer cases and 446 cancer‐free female controls using quantitative polymerase chain reaction with TaqMan probes. To estimate the association between *MALAT1* polymorphisms (rs591291 C>T, rs664589 C>G, and rs4102217 G>C) and the risk of endometrial cancer, an unconditional logistic regression model was conducted to calculate the odds ratio (OR) and the 95% confidence interval (CI), adjusting for surgery history, menopause, number of deliveries, BMI, and FIGO stage.

**Results:**

We found that the *MALAT1* rs664589 C>G polymorphism was significantly associated with endometrial cancer risk (heterogeneous: adjusted OR = 0.57, 95% CI = 0.34‐0.93, *P* = .026; homogenous: adjusted OR = 3.74, 95% CI = 1.12‐12.45, *P* = .032; and recessive: adjusted OR = 4.06, 95% CI = 1.22‐13.48, *P* = .022). Stratified analysis further demonstrated that the *MALAT1* rs664589 C>G polymorphism significantly increased the risk of endometrial cancer susceptibility in patients with no history of surgery, more deliveries, BMI between 25 and 29.9, and FIGO stages II‐III. Compared with the wild‐type GCG haplotype carriers, individuals with CGG haplotypes had a higher risk of developing endometrial cancer.

**Conclusion:**

The *MALAT1* rs664589 C>G polymorphism was associated with a significant increase in endometrial cancer risk.

## 1 INTRODUCTION

Endometrial cancer (EC) is the most common cancer among females worldwide.^1^ The incidence of EC has seen a significant increase in China.^2^ Unopposed estrogen, early menarche, endometriosis, obesity, diabetes, late menopause, hypertension, and nulliparity are now well established as causative agents responsible for endometrial cancer.^3^ Although these risk factors are strongly associated with the risk of endometrial cancer, only a small number of women eventually develop endometrial cancer, suggesting that host genetic variations play critical roles in endometrial cancer development.

Long non‐coding RNAs (lncRNAs) are a class of ncRNAs that are more than 200 nucleotides and are involved in several biological processes.^4^, ^5^, ^6^, ^7^, ^8^ Increasing evidence suggests that lncRNAs are associated with tumor genesis, progress, and treatment response.^9^, ^10^

Metastasis associated with lung adenocarcinoma transcript‐1 (*MALAT1*) is a long intergenic non‐coding RNA (lincRNA), consisting of more than 8000 nts and located on chromosome 11q13.^11^, ^12^ Recent studies have shown that abnormal *MALAT1* expression influenced cancer cell proliferation, invasion, and/or metastasis in various tumors, such as breast cancer,^13^ lung cancer,^14^ and gastric cancer.^15^ Therefore, *MALAT1* has been classified as an oncogene.

However, few studies have focused on the association between the genetic variants of *MALAT1* and the risks of EC. In this study, we sought to identify common genetic polymorphisms of *MALAT1* associated with the risk of EC susceptibility in Southern Chinese women.

## 2 MATERIALS AND METHODS

### 2.1 Patients and controls

In the present case‐control study, 249 endometrial cancer patients were enrolled at The Second Affiliated Hospital and Yuying Children's Hospital of Wenzhou Medical University (WMU) from February 2007 to February 2017. The diagnosis of endometrial cancer was confirmed in all cases by histological examination of tissues from biopsy or resected specimens. Cancer‐free controls (n = 446) were also recruited at the same hospital during routine physical examinations, and we excluded participants who had been diagnosed with malignant neoplasms or had a family history of cancers. This study was approved by The Second Affiliated Hospital and Yuying Children's Hospital of WMU, and written informed consent was obtained from all patients.

### 2.2 Polymorphism selection and genotyping

Three potentially functional polymorphisms (rs591291 C>T, rs664589 C>G, and rs4102217 G>C) were selected through the SNPinfo (http://snpinfo.niehs.nih.gov/) and dbSNP database (http://www.ncbi.nlm.nih.gov/) (Supplemental Table 1). As shown in Supplemental Figure 1, there is no significant linkage disequilibrium (R2<0.8) among these three included SNPs for Chinese Han subjects.The TIAN quick FFPE DNA Kit (Qiagen Inc) was applied to extract genomic DNA from all patients from paraffin‐embedded tissues, while genomic DNA from the controls was extracted from peripheral blood specimens using the TIANamp Blood DNA Kit (TianGen Biotech Co. Ltd.) as described previously.^16^, ^17^The AUV absorption spectrophotometer was used to detect DNA purity and concentration (NanoDrop Technologies Inc).

Genotyping analysis was performed by real‐time PCR with TaqMan PCR master mix and ABI Prism 7900HT genetic detection system. The details of the analysis procedures have been previously described.^18^ In addition, approximately 5% of the samples were randomly selected as positive controls and negative controls for assessing the accuracy of the genotyping results.

### 2.3 Statistical analysis

The goodness‐of‐fit chi‐square test was adopted to evaluate departure from Hardy‐Weinberg equilibrium (HWE) for the selected polymorphisms in control subjects. The heterogeneity of the genotypes and ages between patients and controls was evaluated by using a two‐sided chi‐square test. The association between *MALAT1* polymorphisms and endometrial cancer risk was assessed by an unconditional logistic regression model, calculated as crude and adjusted odds ratio (OR) and 95% confidence interval (CI). Additionally, stratified analyses were performed by surgery history, menopause, number of deliveries, BMI, and FIGO stage. All statistical tests were carried out by SAS software (version 9.4; SAS Institute), with a two‐sided *P*‐value < .05 considered statistically significant.

## 3 RESULTS

### 3.1 Characteristics of the study participants

In the present study, we enrolled 249 endometrial cancer patients with an average age of 54.60 ± 9.09 months and 446 cancer‐free controls with an average age of 53.03 ± 10.84 months. The frequency distributions of selected demographic characteristics of the cases and controls are shown in Table 1. There was no significant difference between cases and controls in age (*P* = .053), surgery history (*P* = .949), menopause (*P* = .075), number of deliveries (*P* = .110), and BMI (*P* = .063).

**Table 1:** Frequency distribution of selected variables for endometrial cancer cases and cancer‐free controls

Characteristic	Case (N = 249)	Control (N = 446)	*P* a
	No. (%)	No. (%)	
Age			.053
Age range, y	26‐85	22‐75	
Mean ± SD	54.60 ± 9.09	53.03 ± 10.84	
Surgery history			.949
Yes	53 (21.29%)	94 (21.08%)	
No	196 (78.71%)	352 (78.92%)	
Menopause			.075
Yes	165 (66.27%)	265 (59.42%)	
No	84 (33.73%)	181 (40.58%)	
Delivery			.110
=0	6 (2.41%)	4 (0.90%)	
=1	62 (24.90%）	98 (21.97%)	
≥2	181 (72.69%)	344 (77.13%)	
BMI			.063
<18.5	21 (8.57%)	27 (6.18%)	
18.5 ≤ BMI ≤ 24.9	113 (46.12%)	248 (56.75%)	
25.0 ≤ BMI ≤ 29.9	89 (36.33%)	143 (32.72%)	
30.0 ≤ BMI ≤ 40	22 (8.98%)	19 (4.35%)	
FIGO stage			
I	219 (87.95%)		
II	9 (3.61%)		
III	21 (8.43%)		

a Two‐sided chi‐square test for distributions between endometrial cancer cases and cancer‐free control.

In terms of the FIGO stage, 219 (88.0%), 9 (3.6%), and 21 (8.4%) cases were distributed into stages I, II, and III, respectively.

### 3.2 Association between selected *MALAT1* polymorphisms and endometrial cancer risk

We investigated the association with endometrial cancer of each of the three selected *MALAT1* polymorphisms. First, the HWE test was used to identify genotyping errors in cancer‐free controls. As shown in Table 2, all the genotype distributions were in accordance with HWE (*P* = .255 for rs591291, *P* = .853 for rs664589, *P* = .720 for rs4102217). Next, single‐locus analysis indicated that *MALAT1* rs664589 C>G was significantly associated with endometrial cancer risk (heterogeneous: adjusted OR = 0.57, 95% CI = 0.34‐0.93, *P* = .026; homogenous: adjusted OR = 3.74, 95% CI = 1.12‐12.45, *P* = .032; and recessive: adjusted OR = 4.06, 95% CI = 1.22‐13.48, *P* = .022). However, other genetic associations with endometrial cancer risk were not discovered in the present study. When the three SNPs were combined, we found that carriers of 2‐3 genotypes did not have a significantly increased risk of endometrial cancer at an OR of 1.25 (95% CI = 0.85‐1.84, *P* = .263).

**Table 2:** Association between selected polymorphisms and endometrial cancer by logistic regression analyses

Genotype	Cases (N = 183)	Controls (N = 603)	*P* a	Crude OR (95% CI)	*P*	Adjusted OR (95% CI)^b^	*P* b
*MALAT1* rs591291 C>T, HWE = 0.255							
CC	90 (36.1%)	153 (34.5%)		1.00		1.00	
CT	123 (49.4%)	225 (50.7%)		0.93 (0.66‐1.31)	.673	0.90 (0.63‐1.28)	.550
TT	36 (14.5%)	66 (14.9%)		0.93 (0.57‐1.50)	.759	0.95 (0.58‐1.54)	.822
Additive			.696	0.96 (0.76‐1.02)	.696	0.96 (0.76‐1.21)	.704
Dominant	159 (63.9%)	291 (65.6%)	.656	0.93 (0.67‐1.29)	.656	0.91 (0.65‐1.27)	.575
Recessive	213 (85.2%)	378 (85.2%)	.885	0.97 (0.62‐1.50)	.885	1.01 (0.65‐1.57)	.977
*MALAT1* rs664589 C>G, HWE = 0.853							
CC	214 (85.9%)	369 (82.7%)		1.00		1.00	
CG	26 (5.2%)	73 (16.4%)		**0.61 (0.38‐0.99)**	**.046**	**0.57 (0.34‐0.93)**	**.026**
GG	9 (9.2%)	4 (0.9%)		**3.88 (1.18‐12.74)**	**.026**	**3.74 (1.12‐12.45)**	**.032**
Additive			.884	0.97 (0.68‐1.40)	.886	0.94 (0.64‐1.36)	.722
Dominant	35 (14.4%)	77 (17.3%)	.270	0.78 (0.51‐1.21)	.271	0.74 (0.47‐1.15)	.179
Recessive	240 (91.1%)	442 (99.1%)	**.011**	**4.14 (1.26‐13.59)**	**.019**	**4.06 (1.22‐13.48)**	**.022**
*MALAT1* rs4102217 G>C, HWE = 0.720							
GG	176 (70.7%)	327 (73.5%)		1.00		1.00	
CG	68 (27.3%)	110 (24.7%)		1.15 (0.81‐1.64)	.443	1.11 (0.77‐1.59)	.583
CC	5 (2.0%)	8 (1.8%)		1.16 (0.37‐3.60)	.796	1.24 (0.40‐3.90)	.708
Additive			.443	1.13 (0.83‐1.54)	.442	1.11 (0.81‐1.52)	.523
Dominant	73 (29.3%)	118 (26.5%)	.429	1.15 (0.81‐1.62)	.428	1.12 (0.79‐1.58)	.543
Recessive	244 (98.0%)	437 (98.2%)	.845	1.12 (0.36‐3.46)	.845	1.21 (0.39‐3.78)	.743
Combined effect of risk genotypes^c^							
0‐1	192 (77.11%)	362 (81.72%)		1.00		1.00	
2‐3	57 (22.89%)	81 (18.28%)		1.33 (0.91‐1.94)	.146	1.25 (0.85‐1.84)	.263

Abbreviations: CI, confidence interval; HWE, Hardy‐Weinberg equilibrium; OR, odds ratio.

a Chi‐square test for genotype distributions between endometrial cancer patients and controls.

b Adjusted for surgery history, menopause, delivery, and BMI.

c Risk genotype was with rs591291 CC, rs664589 GG, and rs4102217 CG/CC.

The results were in bold, if the 95% CI excluded 1 or *P* < .05.

### 3.3 Stratified analysis

We further explored the association of *MALAT1* rs664589 C>G polymorphisms with endometrial cancer susceptibility by stratified analysis. The rs664589 CG/CC genotypes significantly increased endometrial cancer susceptibility in the women with no surgery history, more deliveries, BMI between 25.0 and 29.9, and FIGO stages II‐III (Table 3).

**Table 3:** Stratification analysis of risk genotypes with endometrial cancer susceptibility

Variables	rs591291 (cases/controls)		AOR (95% CI)^a^	*P* a	rs664589 (cases/controls)		AOR (95% CI)^a^	*P* a	rs4102217 (cases/controls)		AOR (95% CI) a	*P* a
	AA	AG/GG			CG/CC	GG			AA/AC	CC		
Surgery history												
Yes	45/77	8/17	0.81 (0.32‐2.02)	.644	50/92	3/2	2.76 (0.45‐17.06)	.275	52/92	1/2	0.89 (0.08‐9.99)	.921
No	168/301	28/49	1.02 (0.62‐1.69)	.927	190/350	6/2	**5.53 (1.11‐27.64)**	**.037**	192/345	4/6	1.20 (0.33‐4.30)	.782
Menopause												
Yes	141/228	24/37	1.05 (0.60‐1.83)	.866	158/2657	7/0	–	–	164/258	1/6	0.26 (0.03‐2.20)	.217
No	72/150	12/29	0.86 (0.42‐1.79)	.690	82/177	2/4	1.08 (0.19‐6.02)	.929	80/179	4/2	4.48 (0.80‐24.93)	.087
Delivery												
=0	5/4	1/0	–	–	6/4	0/0	–	–	6/4	0/0	–	–
=1	57/5	85/13	0.57 (0.19‐1.70)	.315	60/97	2/1	3.23 (0.29‐36.43)	.342	62/97	0/1	–	–
≥2	151/289	30/53	1.08 (0.66‐1.77)	.748	174/341	7/3	**4.57 (1.17‐17.90)**	**.029**	176/336	5/7	1.36 (0.43‐4.36)	.601
BMI												
<18.5	18/26	3/1	4.33 (40.42‐45.06)	.220	21/27	0/0	–	–	21/27	0/0	–	–
18.5 ≤ BMI ≤ 24.9	96/204	17/43	0.84 (0.46‐1.55)	.677	111/246	2/2	2.22 (0.31‐15.93)	.429	112/241	1/6	0.36 (0.04‐3.01)	.345
25.0 ≤ BMI ≤ 29.9	76/123	13/19	1.11 (0.52‐2.37)	.793	83/141	6/2	**5.10 (1.01‐25.83)**	**.049**	86/141	3/2	2.46 (0.40‐15.01)	.330
30.0 ≤ BMI ≤ 40	19/16	3/3	0.84 (0.15‐4.76)	.846	21/19	1/0			21/19	1/0	–	–
FIGO stage												
I	190/378	23/378	0.90 (0.56‐1.45)	.675	211/422	8/4	**4.00 (1.18‐13.61)**	**.027**	214/437	5/8	1.40 (0.45‐4.35)	.566
II	29/66	7/66	1.82 (0.72‐4.57)	.203	29/422	1/4	**15.4 (1.06‐224.76)**	**.046**	30/437	0/8	–	–

Abbreviations: AOR, adjusted odds ratio; CI, confidence interval.

a Adjusted for surgery history, menopause, delivery, and BMI.

The results were in bold, if the 95% CI excluded 1 or *P* < .05.

### 3.4 *MALAT1* haplotypes and endometrial cancer risk

As shown in Table 4, *MALAT1* haplotypes were available for analysis. When compared to the reference haplotype CCG, CGG was associated with significantly increased endometrial cancer risk (adjusted OR = 2.80, 95% CI = 1.00‐7.86, *P* = .050).

**Table 4:** The frequency of inferred haplotypes of *MALAT1* based on observed genotypes and their association with the risk of endometrial cancer

rs591291	rs664589	rs4102217	Cases (N = 555)	Controls (N = 535)	Crude OR (95% CI)	*P*	Adjusted OR (95% CI)	*P* a
C	C	G	244 (49.0%)	442 (49.9%)	1.00		1.00	
T	C	G	132 (26.5%)	240 (27.1%)	1.00 (0.77‐1.30)	.978	0.99 (0.76‐1.30)	.948
C	G	G	10 (2.0%)	6 (0.7%)	**3.02 (1.08‐8.41)**	**.035**	**2.80 (1.00‐7.86)**	**.050**
C	G	C	0 (0.0%)	0 (0.0%)	–	–	–	–
T	G	G	34 (6.8%)	74 (8.4%)	0.83 (0.54‐1.29)	.408	0.79 (0.50‐1.24)	.300
T	G	C	0 (0.0%)	1 (0.1%)	–	–	–	–
C	C	C	49 (9.8%)	81 (9.1%)	1.10 (0.74‐1.62)	.644	1.03 (0.70‐1.53)	.874
T	C	C	29 (5.8%)	42 (2.7%)	1.25 (0.76‐2.06)	.379	1.28 (0.78‐2.13)	.332

Abbreviations: CI, confidence interval; OR, odds ratio.

a Adjusted for surgery history, menopause, delivery, and BMI.

The results were in bold, if the 95% CI excluded 1 or *P* < .05.

## 4 DISCUSSION

In recent years, increasing evidence has indicated that lncRNAs play a crucial role in regulating gene expression in normal cells. MiRNAs have been identified in relation to various biological processes and in the development and progression of diseases.^19^ *MALAT1* is one of the most abundant and well‐conserved lncRNAs, and it has been shown to play an important role in many cancers.

Zhao et al^20^ analyzed *MALAT1* expression in endometrial cancer in 2014. They examined the MALAT1 expression in 32 EEC samples and 9 normal tissues by In situ hybridization (ISH) on the paraffin sections using a MALAT1‐specific probe. As expected, the *MALAT1* signal was highly expressed in EC samples. The authors found that miR‐200c and *MALAT1* expressions were inversely associated in EC cells. In many malignancies, the miR‐200 family is downregulated, which increases the invasive and migratory capacity of tumor cells.^6^ High expression of *MALAT1* affects the progression of cancer. In a previous study, we demonstrated that the expression of *MALAT1* was negatively correlated with that of miR‐200c in ectopic endometrial specimens. Further studies showed that the level of miR‐200c in primary endometrial stromal cells was increased by knocking down *MALAT1*.^21^

Rs591291 is a variant of C/T in the exon region of the *MALAT1* gene. Che et al^22^ found no significant relationship between rs591291 and recurrent miscarriage risk. In our data, we found that there was no relationship in the main effect analysis. Wu et al showed^23^ that *MALAT1* rs664589 C>G was associated with a significantly increased risk of colorectal cancer. The authors demonstrated that *MALAT1* with the rs664589 G allele had altered binding to miR‐194‐5p in the nucleus, leading to increased *MALAT1* expression and enhanced colorectal cancer development. Other studies noted that rs664589 polymorphisms were not associated with the risk of esophageal squamous cell carcinoma^24^ or coronary atherosclerotic heart disease.^25^ We found that the *MALAT1* rs664589 C>G polymorphism was significantly associated with increased endometrial cancer risk. This polymorphism could be used as a genetic locus to predict cancer. Hu et al^26^ demonstrated that the GC genotype and the recessive model of rs4102217 potentially increased coronary atherosclerotic heart disease risk in some specific groups. *MALAT1* 4102217 C>G association with endometrial cancer risk was not discovered in our study.

Although the current case‐control study provided evidence that the common *MALAT1* polymorphisms are associated with the risk of endometrial cancer susceptibility in Southern Chinese women, several limitations should be addressed. First, only three common *MALAT1* polymorphisms were genotyped in the current study; hence, additional common *MALAT1* polymorphisms should be investigated to fully illuminate the contribution of polymorphisms in *MALAT1* to endometrial cancer susceptibility. Second, the sample size in the current study was still relatively small. Additionally, selecting samples from one hospital in the current hospital‐based case‐control study may result in selection bias and even decreased or increased risk assessment. Third, other than polymorphisms, many confounders could also influence the susceptibility to endometrial cancer, such as gene‐gene interactions, gene‐environment interactions, and specific tumor pathologic classification, and were not taken into consideration due to lack of individual patient information. Fourth, although our findings suggested that *MALAT1* rs664589 C>G significantly increased the risk of endometrial cancer, more studies are needed to confirm this result and determine potential mechanisms and functions in the future.

## 5 CONCLUSION

The present study provides evidence that *MALAT1* rs664589 C>G significantly increased the risk of endometrial cancer. These findings need to be verified or linked with functional studies.

## AUTHOR CONTRIBUTIONS

PD designed the study; MYZ, ZWL, SLC, FC, JWZ, and MMZ collected the samples and information; GGC and JH performed the experiments and analyzed the data; GGC and JH wrote and edited the article.
