# Supplementary material for: Association of polymorphisms in MALAT1 with the risk of endometrial cancer in Southern Chinese women
Source: J Clin Lab Anal. 2019 Dec 27;34(4):e23146. doi: 10.1002/jcla.23146 (PMC7171330; doi:10.1002/jcla.23146)

| **Supplemental Table 1. Potential functional polymorphisms in MALAT1 genes as predicted by SNPinfo online software** | | | | | | | | | | | | | | |
| --- | --- | --- | --- | --- | --- | --- | --- | --- | --- | --- | --- | --- | --- | --- |
| **rs** | **Chr** | **Allele** | **Position** | **TFBS** | **Splicing**  **(ESE or ESS)** | **Splicing**  **(abolish domain)** | **miRNA**  **(miRanda)** | **miRNA**  **(Sanger)** | **nsSNP** | **Nearby Gene** | **Distance (bp)** | **Allele** | **Asian** | **CHB** |
| rs4102217 | 11 | C/G | 65020471 | Y | -- | -- | -- | -- | -- | *MIRN612\|\|MALAT1* | -51868\|\|-1939 | G | 0.994 | 1 |
| rs591291 | 11 | C/T | 65021058 | Y | -- | -- | -- | -- | -- | *MIRN612\|\|MALAT1* | -52455\|\|-1352 | - | -- | -- |
| rs664589 | 11 | C/G | 65025925 | -- | -- | -- | -- | Y | -- | *MALAT1* | 3515\|\|5074 | C | 0.915 | 0.867 |

Figure S1 Linkage disequilibrium analysis for the 3 selected polymorphisms in malat1 gene in Han Chinese population consisting of CHB (Han Chinese in Beijing, China) and CHS (Southern Han Chinese) subjects.
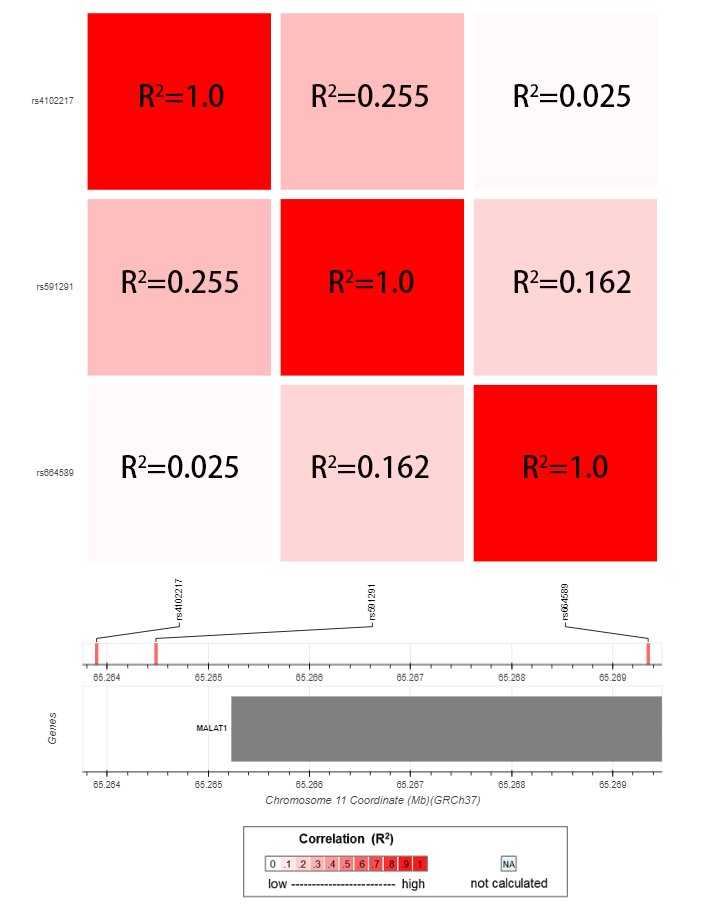

Supplement: Supplementary file 1 [file JCLA-34-e23146-s001.docx]
